# Assessment the impact of different fuels used in cement industry on pollutant emissions and ambient air quality: a case study in Egypt

**DOI:** 10.1007/s40201-022-00844-9

**Published:** 2022-11-30

**Authors:** Tarek Sayad, Fawzia Ibrahim Moursy, Attia M. El-Tantawi, Mohamed Saad, Mostafa Morsy

**Affiliations:** 1 Astronomy and Meteorology Department, Faculty of Science, Al-Azhar University, 11884 Cairo, Egypt; 2 Department of Natural Resources, Faculty of African Postgraduate Studies, Cairo University, 12613 Giza, Egypt; 3 Air Quality and Noise Department, Egyptian Environmental Affairs Agency, Ministry of Environment, Maadi, Cairo, 11728 Egypt

**Keywords:** Different fuel types, Titan Cement Company, Impact assessment, AERMOD dispersion model, Pollutants, Air quality, Egypt

## Abstract

This study aims to assess the impact of using different fuels in Egyptian Titan Alexandria Portland Cement Company on emissions and concentrations of pollutants (Total suspended particles (TSP), nitrogen dioxide (NO_2_‎), and sulfur dioxide (SO_2_)) and their influence on ambient air quality during the period 2014–2020 using AERMOD dispersion model. The results showed that changing the fuel from natural gas in 2014 to coal mixed with alternative fuels (Tire-Derived Fuel (TDF), Dried Sewage Sludge (DSS), and Refuse Derived Fuels (RDF)) in 2015–2020 caused fluctuating variations in pollutant emissions and concentrations. The highest and lowest maximum concentrations of TSP occurred in 2017 and 2014 respectively, where the TSP is positively correlated with coal, RDF, and DSS and negatively correlated with natural gas, diesel, and TDF. Also, the lowest and highest maximum NO_2_ concentrations were detected in 2020 and 2016 followed by 2017 respectively, where NO_2_ is positively correlated with DSS and negatively correlated with TDF and varies with diesel, coal, and RDF. Moreover, the maximum concentrations of SO_2_ were the lowest in 2018 and highest in 2016 followed by 2017 because of its considerable positive correlation with natural gas and DSS and negative correlation with RDF, TDF, and coal. Generally, it was found that increasing the percentage of TDF and RDF with decreasing the percentage of DSS, diesel, and coal will reduce pollutant emissions and concentrations and enhance ambient air quality.

## Introduction

The cement industry in Egypt has witnessed a steady growth since 1927 after the establishment of the Tora Portland Cement Company [33]. The cement industry in Egypt ranks twelfth in the world in terms of economic and vital strength that supports the construction sector, which represents about 5% of GDP for Egypt [18] and represents one of the most important global strategic industrial sectors [5]. The cement industry is one of the most energy consuming industries in the world [17, 21] as it is one of the most important sources of human-caused air pollution that affects air quality [2] which contributes about 5–8% of carbon dioxide emissions [6, 24], while it contributes about 40% of the total greenhouse gas emissions from all industrial sectors in Egypt [25]. Cement manufacturing produces many types of pollutions such as dust (PM10 and PM2.5), nitrogen oxides (NO_x_), sulfur dioxide (SO_2_), carbon monoxide (CO), carbon dioxide (CO_2_) and small quantities of volatile organic compounds, ammonia, chlorine, hydrogen chloride as well as some heavy metals [2, 3, 10, 26]. The high emissions of SO_2_ and dust are produced due to the use of coal in the steel, aluminum and cement industries, while the high emission of NO_x_ is due to the use of diesel [16]. SO_2_ negatively affects air quality and increases risks to human health [29], such as difficulty breathing, corneal haze, bronchitis, psychological changes, and heart failure [9, 20, 30]. Therefore, about 4.2 million people die worldwide per year in 2016 as a result of exposure to ambient air pollution [31].

Energy costs and environmental concerns have encouraged cement companies in Egypt to assess the potential for replacing conventional fuels with alternative fuels (Refuse Derived Fuels (RDF), Dried Sewage Sludge (DSS), agricultural waste, and Tire-Derived Fuel (TDF)). The use of alternative fuels affects pollutant emissions and therefore it is necessary to plan and promote various methods that can reduce the environmental impacts and economic costs of the cement industry. Recently, to reduce the potential energy consumption in the cement manufacturing process and reduce greenhouse gas emissions, several research studies have been developed to introduce sustainable raw materials or alternative fuels into the cement industry (e.g., [28, 32]). The Egyptian cement factories continued to use natural gas as fuel until 2014, but by 2015 the availability of natural gas to cement companies became uncertain, forcing companies to explore alternative energy sources including imported coal and coke. Meteorological factors, atmospheric stability, stack height, and topography interact in complex ways that greatly influence the spatio-temporal distribution, transport and dispersion of air pollution [12, 14]. Pollutant dispersion modeling is a good and appropriate tool for simulating these complex interactions, which depends on the data of many different elements, whether natural or man-made, to simulate the concentration, dispersion, and distribution of air pollutants over large distances emitted from various sources (e.g., cement plants). Several advanced dispersion models, like AERMOD, can use meteorological data obtained from the output of numerical weather prediction (NWP) models using the pre-processing module.

Therefore, the main objectives of this study are: (i) investigate the influence of using different alternative fuels in Egyptian Titan Alexandria Portland Cement Company (APCC) on emissions and concentrations of pollutants (dust, NO_2_, and SO_2_) and their impact on ambient air quality; (ii) Determine the most influential fuels that have a strong correlation (negative or positive) with emissions and concentrations of pollutants dispersed around APPC during the period from 2014 to 2020; (iii) Compare the simulated highest maximum concentrations of pollutants with the standards of the Egyptian Environmental Affairs Agency (EEAA) and the estimated in the base year (2014); and (iv) Interpret and analyze the spatial distribution of maximum concentrations of pollutants around the APCC at 1-hour, 24-hours, and 1-year time-scales across the study period.

## Data and methodology

### Applicable local laws and regulations for emission levels

The Egyptian Environmental Affairs Agency (EEAA) has the powers to set the local legal limits, regulations, standards, and conditions for environmental protection, monitor the compliance of industrial units, and take actions against violators. In 1994, EEAA issued the main Environmental Law No. 4, where the applicable Egyptian air quality standards relevant to this study were listed in Appendix No. 5 of the executive regulations in this law, which was amended by Law No. 9 of 2009. These standards represent the maximum permissible concentration within one hour, 24 h and one year as shown in Table 1.

**Table 1:** The maximum permissible limits for basic air pollutants according to Appendix 5 in the EEAA executive regulations Law No. 4 of 1994, which was amended by Law No. 9 of 2009

Pollutant	Maximum concentration µg/m^3^		
	1-hour	24-hours	1-year
Total suspended particles (TSP)	-----	230	125
Nitrogen dioxide (NO_2_)	300	150	80
Sulfur oxides (SO_2_)	350	150	60

### Climate of the study area

**Fig. 1:**
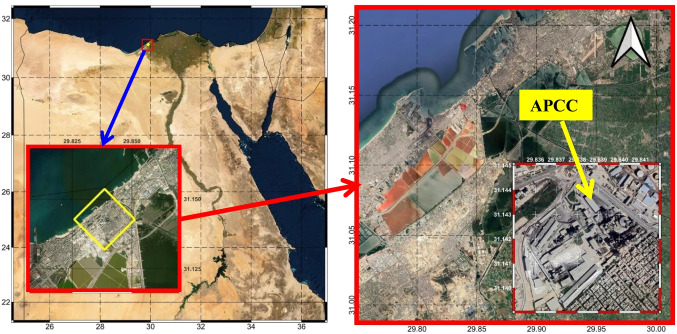
Location of Titan Alexandria Portland Cement Company (APCC)

Titan Alexandria Portland Cement Company (APCC) is located inside Wadi Al-Qamar region, west of Alexandria Governorate, on the Mediterranean coast of Egypt at 31.14° N and 29.84° E at an altitude of 5 m above mean sea level as shown in Fig. 1. The regional climate in Alexandria is relatively mild with variation in rainfall and temperature characteristics throughout the year due to its location on the Mediterranean Sea. According to Egyptian Meteorological Authority report [8], the average annual temperature in Alexandria is 20.43° C, where August (January) is the warmest (coldest) month in across the ‎year with average temperature of 27.06° (13.83°) C. The difference between the ‎hottest month and the coldest month is 13.23° C. Moreover, the annual amount of rainfall in Alexandria is 192.65 mm, with the largest amount of precipitation being 167.5 mm (about 87% of the annual amount) during the winter season (November to March). Recently, there has been a change in rainfall intensity and the distribution of rainy days, as the frequency of rainfall has decreased (i.e., few rainy days across the year) and the amount of water has increased during a single storm [19].

### APCC emission monitoring system and fuels used

APCC is chosen in this study because it represents one of the most important and largest cement plants in Egypt that is concerned with applying local legal limits and regulations for environmental protection as one of the sustainable development goals. It also implements an online data monitoring system using SICK’s MCS100E HW multicomponent analyzer systems to measure and report air emissions in order to ensure complete control and reduce the environmental impacts resulting from cement production. The gas device is automatically calibrated once a day using inside reference cells, and it also calibrated by the company every three months using special standard gasses calibration cylinders, while the dust monitor is calibrated once every three months. The APCC works 24 h a day, 7 days a week (24 h/7 day), with a continuous monitoring system for emissions from the main chimney of the APCC. These measurements are transferred directly to the EEAA control room which enables to monitor the emissions closely in real time and take actions accordingly. Table 2 shows the percentages of different fuels used to operate the APCC during the available study period from 2014 to 2020.

**Table 2:** Percentage of alternative fuels used for the APCC plant during the period 2014–2020

Type of fuel used	2014	2015	2016	2017	2018	2019	2020
Natural gas	97.3	0.8	0.9	0.67	0.28	------	------
Diesel	2.7	42.3	23.27	1.91	1.55	2.1	1.3
Coal	------	33.6	70.99	83.36	81.01	78.4	81.6
RDF	------	23.3	4.28	14.05	17.16	16.8	12.2
DSS	------	------	0.56	0.01	------	------	0.1
TDF	------	------	------	------	------	2.7	4.8
Total Percentage %	100	100	100	100	100	100	100

During 2014, APCC relied only on both natural gas and diesel with percentages of 97.3% and 2.7% respectively, so it will be considered as a reference year. Then, natural gas was replaced by other fuels such as coal, Refuse Derived Fuels (RDF), Dried Sewage Sludge (DSS) and Tire-Derived Fuel (TDF) with various percentages during the years from 2015 to 2020 according to Table 2. The percentages of the used fuel types were nearly equal in 2015, while the used coal exceeds 70% during 2016–2020 and the natural gas reduced to less than 1% during 2015–2018 and not used 2019 and 2020. Accordingly, the percentages of change in emissions can be determined according to the change in the different type of fuel compared to 2014 as the base year.

### AERMOD model description

**Fig. 2:**
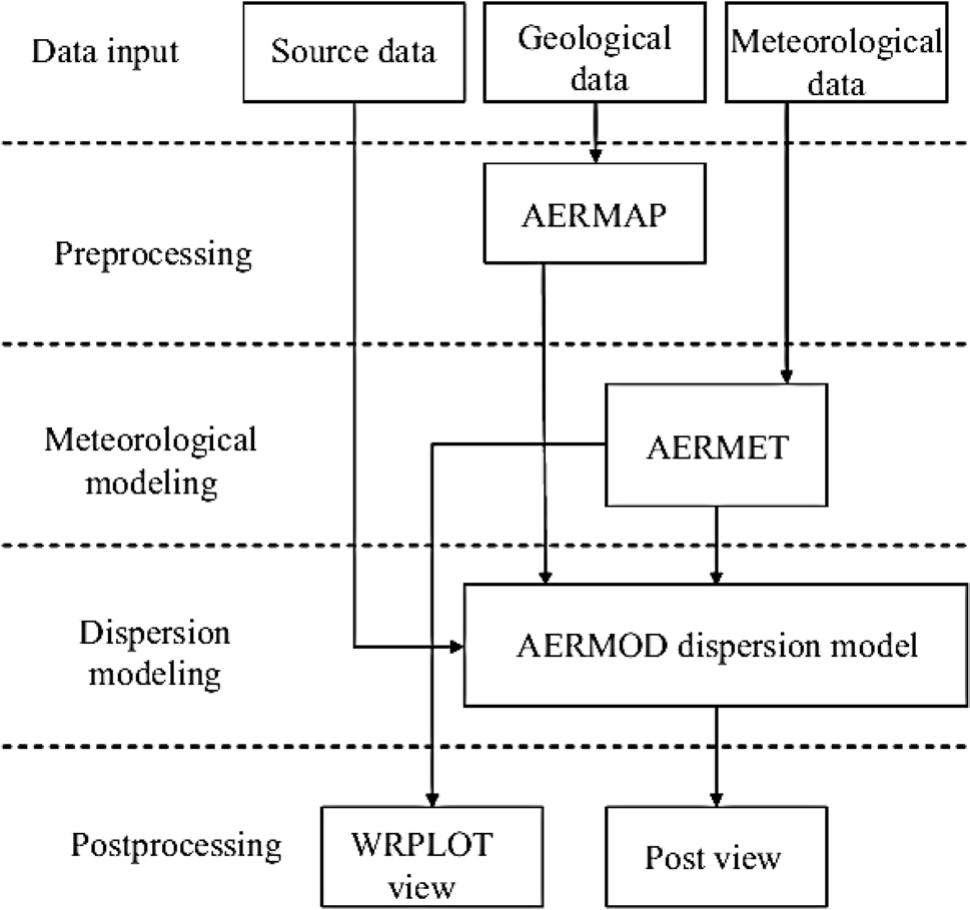
Flowchart for the AERMOD modeling system. (source: Seangkiatiyuth et al. [23])

The state-of-the-art AERMOD Gaussian plume model version 21,112 is used to achieve the purposes of this study. AERMOD was introduced and developed by the American Meteorological Society (AMS)/Environmental Protection Agency (EPA) Regulatory Model Improvement Committee (AERMIC) based on a new basis to be used for regulatory purposes. AERMOD is the preferred and approved air dispersion model by the U.S. EPA in conjunction with the AMS (EPA-454/R-03-004). AERMOD is one of the most widely used regulatory models in the world that incorporate air dispersion based on more complex and advanced algorithms and concepts for each of the planetary boundary layer (PBL) and the terrain. According to US EPA [27], the AERMOD model is accurate for simulating air dispersion at distances of no more than 50 km from the emission source. Inside the AERMOD model there are four main modules; (i) AERMOD Meteorological Preprocessor (AERMET), which prepares meteorological data files for both the surface and the upper air vertical profile; (ii) AERMOD Terrain Preprocessor (AERMAP), which handles the terrain data, layout of receptors, and emission sources to finally generate the AERMOD control files; (iii) the AERMOD Gaussian Plume core (solvers) with the PBL turbulence structure modules; and lastly (iv) AERMOD post-processor (WRPLOT) module, which is used to display the pollutants dispersion maps to perform all related analysis. These four main components of the AERMOD model operate sequentially as illustrated in Fig. 2.

### AERMOD domain and receptors grid layout

**Fig. 3:**
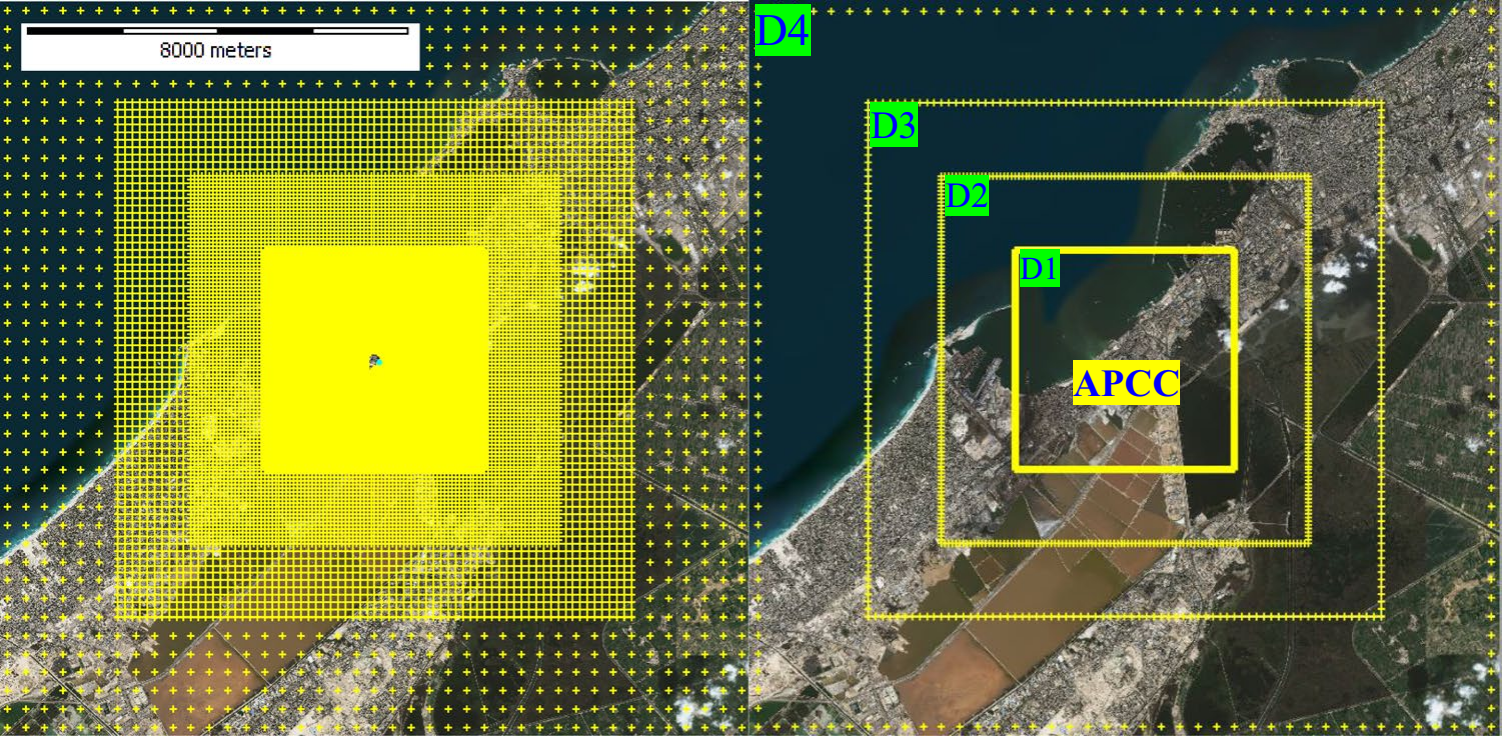
Spatial distribution of AEROMD model receptors within the designed four domains

The AERMOD domain is chosen to cover a wide area (400 km^2^) with a reference point centered on the APCC main stack to capture all spatial distributed downwind pollutant concentration and dispersion. Four restricted domains are specified in one modelling run (multi-tier grid) and graduates from coarse to dens grids (Fig. 3) with total number of 24,016 receptors. The structure of the applied multi-tier grid receptors is explained in Table 3, where the dens receptors are identified for the first (inner) domain (D1) and reduced gradually outward as for the fourth domain (D4).The multi-tier grid receptors type is performed in this study to reduce the required computational time over a large area for a dense receptors network, where the highest pollutants concentration is often dispersed near and around the emission source (APCC main stack).

**Table 3:** Pollutant concentration receptors designed in the four study domains

Domain	Receptors outside inner domain	Coverage area (m)	Grid spacing (m)
First domain (D1)	14,400	6,000 × 6,000	50
Second domain (D2)	6,400	10,000 × 10,000	100
Third domain (D3)	2,400	14,000 × 14,000	200
Fourth domain (D4)	816	20,000 × 20,000	500

### AERMOD input data

AERMOD model requires three different types of data for emission rates, meteorological parameters, and terrain data along with local features. The data used in this study and required to run the AERMOD can be classified as follows:

#### Emissions data

The APCC plant is located at 770731.91 E and 3448886.87 N UTM coordinate into 35R zone and has a main stack height (HS) of 120 m with an inside stack diameter (DS) of 3.15 m. The flow rate or the stack gas exit velocity (VS) and the stack exit temperature (TS) are measured using SICK’s FLOWSIC100 ultrasonic volume flow measurement for continuous emission monitoring. Where, the VS and TS measurements take place continuously across the entire duct diameter and supplies representative measurement results, even with unfavorable flow conditions and the fully automated gain control function of the FLOWSIC100 guarantees signal transmission. Also, the fluctuations in the gas composition, pressure, temperature, and humidity have no influence on the measurement result. The annual average of hourly data for TS,0 VS, and emissions rate (QS) for the emitted TSP dust (Total Suspended Particles) and gas (SO_2_ and NO_2_) during the study period (2014–2020) are shown in Table 4. These parameters are one of the key input variables for AERMOD dispersion plume calculations.

**Table 4:** Annual average of hourly data for TS, VS, and QS (emissions rate) from the main stack of the plant

Parameter	2014	2015	2016	2017	2018	2019	2020
Temperature (°C)	119.92	122.39	118.43	131.13	142.23	128.77	130.27
Gas velocity (m/s)	13.64	19.13	18.91	19.79	21.29	20.36	14.30
TSP dust load (ton/year)	4.37	16.83	31.69	77.81	24.48	21.76	14.46
SO_2_ load (ton/year)	77.45	58.14	83.36	96.34	35.46	56.13	44.18
NO_2_ load (ton/year)	1234.40	2303.54	2010.48	2276.44	1781.51	1312.34	548.25

#### Meteorological data

Meteorological data is one of the major and significant factors required for any pollutant dispersion model. Hourly meteorological (surface and upper air) data for at least one full year for several weather parameters are required in order to characterize the PBL structure over the study area before performing air dispersion. Due to the absence of upper air sounding data and some surface parameters that specify atmospheric stability are also not measured in regular meteorological stations, the MM5 (version 3.6) model is used to provide AERMOD with surface and upper air data at the study location. MM5 is a nonhydrostatic limited-area weather forecasting model developed at Pennsylvania State University and National Center for Atmospheric Research (NCAR) as a community mesoscale model [13]. The initial and boundary condition required to run MM5 is obtained from the National Centers for Environmental Prediction-World Area Forecast System (NCEP-WAFS) dataset every 6 h with 1.25° x 1.25° spatial resolution for the 7-years period 2014–2020. MM5 is carried out for each year separately every 1-hour output at 41 vertical pressure levels using one-way nesting domains. The first and second domains have 36 and 12 km horizontal resolutions respectively, where the second domain (12 km) is centered over Egypt (22–32 °N and 24–37 °E). Whereas the findings of Mass et al. [22] revealed that the reduced 12 km for MM5 output from 36 km has more accurate results for simulating 2 m air temperature, precipitation, sea level pressure and 10 m wind in the Pacific Northwest.

The extracted surface parameters from MM5 12 km output include wind speed, direction, ambient air temperature, total sky cover (cloud cover), station pressure and surface characteristics (albedo, surface roughness, and Bowen ratio). While, the upper air parameters include wind speed, wind direction, atmospheric pressure, and dry bulb temperature at different height levels of 10, 19, 57, 94, 142, 199, 266, 344, 441 and 560 m. These extracted surface and upper air meteorological data files are processed by the AERMET module to reproduce the surface (SFC) and vertical profile (PFL) meteorological files into the recognized AERMOD input format.

#### Terrain and local features data

The AERMAP terrain processor module requires sufficient information about the terrain height which affects the air dispersion as well as the geolocation foreach receptor and source. Thus, the terrain elevation (60-second) from the USGS Digital Elevation Model (DEM) dataset (https://earthexplorer.usgs.gov/) is used along with the layout of receptors and emission sources as input to AERMAP in order to produce the desired AERMOD control file.

Finally, based on these different input data (emission rates, meteorological parameters, and terrain height), AERMOD estimates the 24-hours and 1-year averaged concentrations for TSP, NO_2_, and SO_2_ besides estimating the short term of 1-hour concentration for both NO_2_ and SO_2_ at all receptors using the Gaussian plume formulation.

## Results and discussions

### Wind analysis

**Fig. 4:**
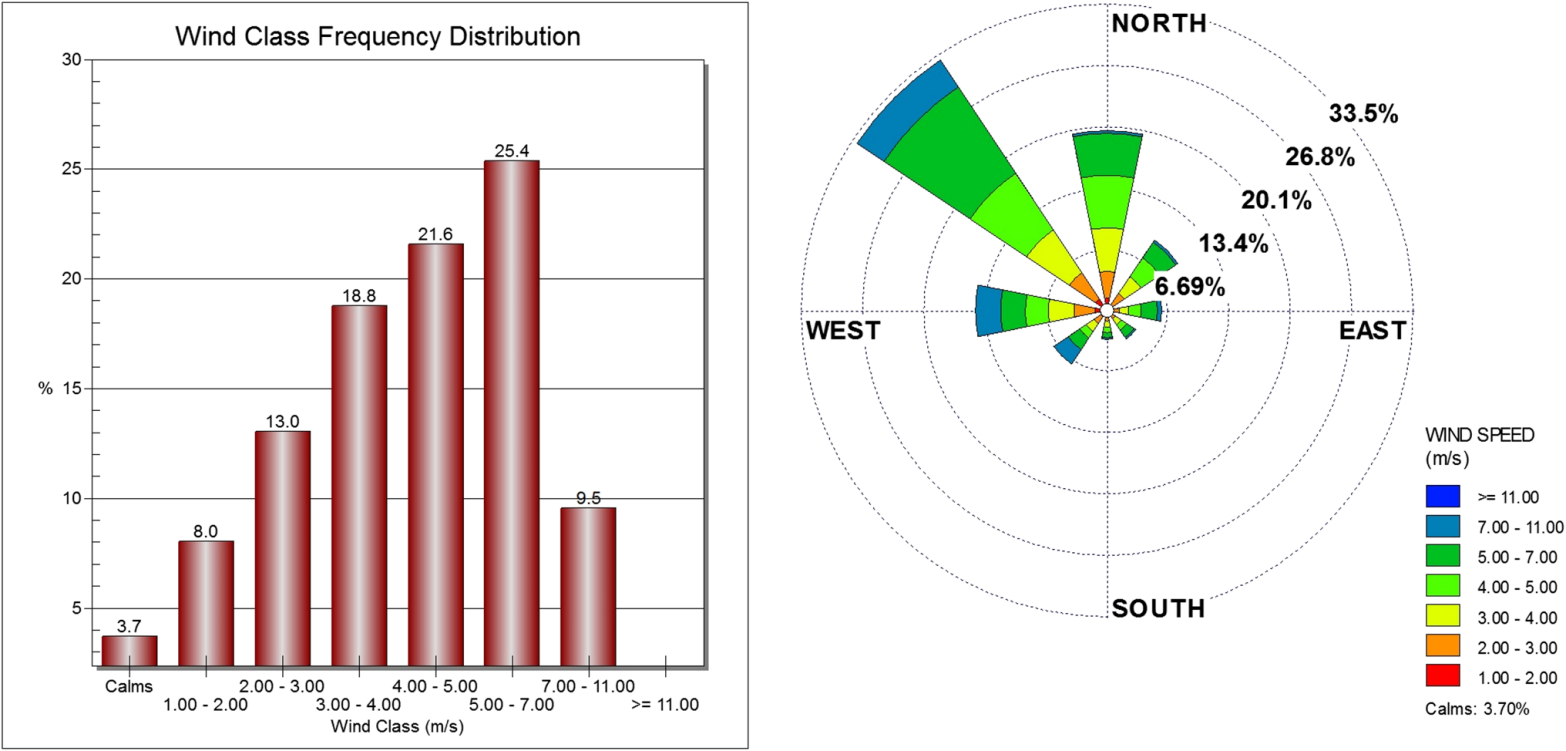
Wind rose diagram (right panel) and wind class frequency distribution (left panel)

The wind rose is analyzed, using WRPLOT VIEW-8.0.2 software, as an average over the selected seven years of study (2014–2020) at APCC site. It is noticed that most of the prevailing winds over the study area are the northwest (32.8%), north (19.6%), west (14.4%) and northeast (9.5%) as shown in Fig. 4. The calm winds represent 3.7% while the average wind speed during the seven years was 4.3 m/s. It is noticed that the wind speed of 4–5 m/s have the highest ratio for north and northeast, while the east, southeast and northwest wind records highest ratio of wind speed 5–7 m/s. Furthermore, the highest wind speed of 3–4 m/s is detected for southerly and westerly wind as well as the highest wind speed of 7–11 m/s is obtained for southwest only.

The frequency distribution of wind speed and direction at APCC for each individual year from 2014 to 2020 indicates that the prevailing winds have the same average frequency distribution behavior over the period 2014–2020. Where, the prevailing wind directions are the northwestern (27.3%-37.1%) and northern (15.7%-20.5%) winds, followed by western (10.5%-13.1%) and northeast (7.1%-11.5%) winds across all study years as shown in Table 5.

**Table 5:** Frequency distribution of wind direction throughout the period 2014–2020

Direction	2014	2015	2016	2017	2018	2019	2020	Average
N	19.4%	15.7%	21.1%	24.1%	18.2%	18.3%	20.5%	19.61%
NE	9.5%	7.1%	9.1%	9.5%	10.6%	11.5%	8.1%	9.34%
E	6.5%	4.0%	5.6%	7.2%	7.3%	6.9%	4.7%	6.03%
SE	4.7%	3.7%	3.9%	3.8%	4.1%	3.7%	3.0%	3.84%
S	4.2%	3.8%	3.3%	2.7%	2.7%	3.3%	2.7%	3.24%
SW	8.1%	7.3%	6.4%	6.2%	6.3%	8.8%	6.5%	7.09%
W	13.0%	20.1%	12.4%	10.5%	15.0%	16.6%	13.1%	14.39%
NW	31.0%	34.5%	35.2%	31.9%	32.4%	27.3%	37.1%	32.77%
Total ratio	96.4%	96.2%	97.0%	95.9%	96.6%	96.4%	95.7%	96.31%
Calm	3.6%	3.8%	3.0%	4.1%	3.4%	3.6%	4.3%	3.69%

### Total suspended particles (TSP) analysis

**Fig. 5:**
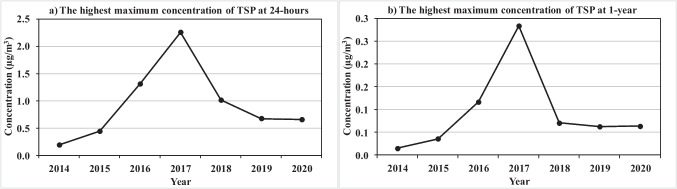
The simulated highest maximum ground level TSP concentration (µg/m^3^) for **a** 24-hours and **b** 1-year

The highest maximum for 24-hours and 1-year TSP ground level concentrations (µg/m^3^) around the APCC as simulated by AERMOD based on the annual change in fuels used over the period 2014–2020 are shown in Fig. 5. The maximum TSP ground level concentrations during the study period for 24-hours is 2.25 µg/m^3^ (Fig. 5a) and for 1-year is 0.28 µg/m^3^ (Fig. 5b) detected in 2017.

All years from 2015 to 2020 have different TSP concentrations and higher than the base year (2014) which has the lowest maximum TSP concentration as illustrated in Table 6. The maximum concentration of TSP increased in 2015 than 2014 by132.03% for 24-hours and 143.24% for 1-year (Table 6) as diesel was used by 42.3%, coal by 33.6%, and RDF by 23.3% (Table 2).

**Table 6:** TSP highest maximum ground level concentrations, distances from the APCC main stack, and change rate from the base year and EEAA standards

TSP	24-hours Concentration				1-year Concentration			
Year	Value (µg/m^3^)	Distance (m)	Change rate (%) from		Value (µg/m^3^)	Distance (m)	Change rate (%) from	
			Base year	EEAA standards			Base year	EEAA standards
2014	0.1892	866	-------	-99.92	0.0148	991	-------	-99.99
2015	0.4390	391	+ 132.03	-99.81	0.0360	821	+ 143.24	-99.97
2016	1.3074	851	+ 591.01	-99.43	0.1168	991	+ 689.19	-99.91
2017	2.2531	973	+ 1090.86	-99.02	0.2841	1059	+ 1819.59	-99.77
2018	1.0099	948	+ 433.77	-99.56	0.0705	1141	+ 376.35	-99.94
2019	0.6733	920	+ 255.87	-99.71	0.0627	1112	+ 323.65	-99.95
2020	0.6535	885	+ 245.40	-99.72	0.0635	953	+ 329.05	-99.95

An increase in the used coal to 70.99% and a decrease in diesel and RDF to 23.27% and 4.28% respectively during 2016 increased the maximum TSP concentrations by 591.01% for 24-hours and 689.19% for 1-year. The increase in the percentage of the used coal (83.36%) with a decrease in the RDF percentage (14.05%) during 2017 increased the maximum concentration levels of TSP. The slight decrease in the percentage of the used coal (81.01%) during 2018 led to reduce the increasing of TSP maximum concentration levels. The entry of TDF fuel with 2.7% percentage during 2019 reduced the increasing of TSP maximum concentration levels although the percentage of the used coal (78.4%) and RDF (16.8%) are still large. Despite the large percentages of the used coal (81.6%) and RDF (12.2%) during 2020, the increase in the percentage of TDF fuel used (4.8%) reduced the increasing of TSP maximum concentration levels compared to the base year. This result is consistent with the study of Alfianto and Lestari [4] which revealed that converting 50% of the used fuels to TDF can reduce the TSP by 41%, in addition to increasing the substitution of fuel with TDF leads to reduced emissions from cement plants. Furthermore, the TSP emission at the main stack and its maximum concentration levels either at 24-hours or 1-year are negatively correlated with natural gas, diesel, and TDF, while it is positively correlated with coal, RDF, and DSS as shown in Table 7. Where, both TSP emission and concentration levels have the highest positive and negative correlations with natural gas and coal respectively. Thus, increasing the percentage of TDF with decreasing the percentage of coal, DSS, RDF will decrease the concentration of TSP and enhance the ambient air quality.

**Table 7:** Correlation between the fuel and the TSP emission and concentration levels across the period 2014–2020

Time-average	Natural gas	Diesel	Coal	RDF	DSS	TDF
24-hour	-0.47	-0.19	+ 0.63*	+ 0.07*	+ 0.23*	-0.26
1-year	-0.38	-0.21	+ 0.53*	+ 0.05*	+ 0.11	-0.21
Emission	-0.42	-0.15	+ 0.53*	+ 0.16	+ 0.06*	-0.28*

* = statistically significant (*p*-value < 0.05)

**Fig. 6:**
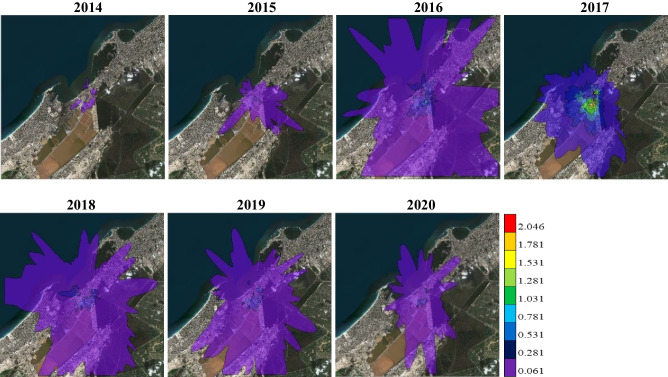
The simulated maximum ground level concentrations (µg/m^3^) of TSP for 24-hours

**Fig. 7:**
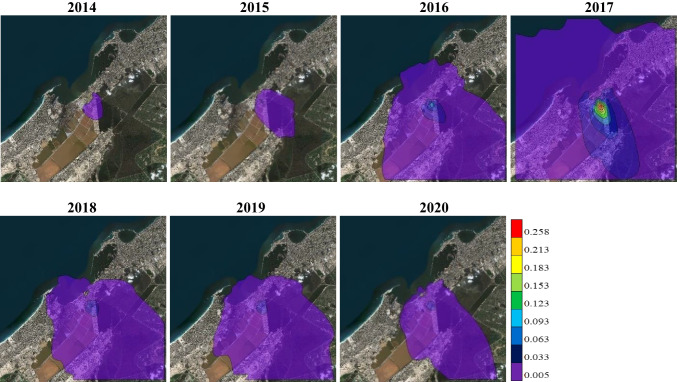
The simulated maximum ground level concentrations (µg/m^3^) of TSP for 1-year

Figures 6 and 7 show the spatial distribution of TSP maximum concentrations dispersed over APCC and its vicinity at time average of 24-hours and 1-year respectively during the period 2014–2020 as simulated by the AERMOD model. It is evident that, for both 24-hours and 1-year, the maximum ground level concentrations of TSP are mostly found southeast and south of the APCC chimney (emission source) and gradually decreased according to the intensity of wind speed and direction. It is noted that the closest receptor of the TSP maximum concentration to the APCC main stack throughout the study years occurred in 2015 for both 24-hours (391 m south) and 1-year (821 m south), because of about 60% of the prevailing winds are less than 4 m/s. On the other hand, the farthest receptor of the TSP maximum concentration is detected during the period from 2017 to 2019 for both 24-hours and 1-year, because more than 50% of the prevailing winds are above 4 m/s and the winds above 7 m/s exceeded 10%.

The maximum concentration and dispersion of TSP is the lowest at both 24-hours and 1-year during 2014 followed by 2015 and 2020, due to the presence of natural gas (97.3%) in 2014, increasing of diesel percentage (42.3%) in 2015, and presence of TDF (4.8%) in 2020. Also, the maximum concentration and dispersion of TSP is the highest at both 24-hours and 1-year due to the presence of coal (83.36%) with DSS (0.01%) during 2017, while it slightly decreased due to the slight decrease of coal (81.01%) in 2018 followed by 2019 due to the presence of TDF (2.7%). Overall, the simulated TSP concentrations at both 24-hours and 1-year have very small values that represent less than 1% from the EEAA standard limits. Additionally, there is no EEAA standard limit for the TSP concentrations at 1-hour time average in the environmental report, so the predicted 1-hour TSP concentrations not discussed.

### NO_2_ and SO_2_ analysis

**Fig. 8:**
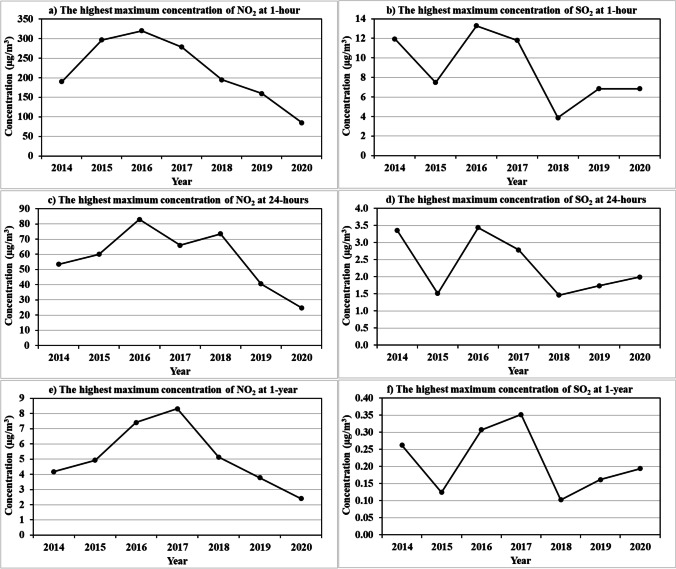
The simulated highest maximum ground level concentrations (µg/m^3^) of NO_2_ (left panels) and SO_2_ (right panels) for **a**-**b** 1-hour, **c**-**d** 24-hours, and **e**-**f** 1-year

Figure 8; Table 8 demonstrate the simulated maximum ground level concentrations of SO_2_ and NO_2_ for 1-hour, 24-hours, and 1-year under the change of the used fuel type during the study period 2014–2020. Also, these maximum concentrations are compared with the base year (2014) and the EEAA standards (Table 8) to assess the impact of the different type of fuel used on variation of these concentrations, which in turn affects the surrounding environment.

**Table 8:** NO_2_ and SO_2_ highest maximum ground level concentrations, distances from the APCC main stack, and change rates from the base year and EEAA standards

Average Time	Year	Distance (m)	NO_2_ Maximum Concentration			SO_2_ Maximum Concentration		
			Value (µg/m^3^)	Change rate (%) from		Value (µg/m^3^)	Change rate (%) from	
				Base year	EEAA standards		Base year	EEAA standards
1-hour	2014	173	190.40	-------	-36.53	11.90	-------	-96.60
	2015	235	296.60	+ 55.78	-1.13	7.50	-36.97	-97.86
	2016	177	320.50	+ 68.33	+ 6.83	13.30	+ 11.76	-96.20
	2017	235	278.90	+ 46.48	-7.03	11.80	-0.84	-96.63
	2018	233	194.70	+ 2.26	-35.10	3.90	-67.23	-98.89
	2019	283	160.00	-15.97	-46.67	6.80	-42.86	-98.06
	2020	211	85.10	-55.30	-71.63	6.90	-42.02	-98.03
24-hour	2014	866	53.40	-------	-64.40	3.35	-------	-97.77
	2015	391	60.10	+ 12.55	-59.93	1.52	-54.63	-98.99
	2016	851	82.90	+ 55.24	-44.73	3.44	+ 2.69	-97.71
	2017	973	65.90	+ 23.41	-56.07	2.79	-16.72	-98.14
	2018	948	73.50	+ 37.64	-51.00	1.46	-56.42	-99.03
	2019	920	40.60	-23.97	-72.93	1.74	-48.06	-98.84
	2020	885	24.80	-53.56	-83.47	2.00	-40.30	-98.67
1-year	2014	991	4.20	-------	-94.75	0.26	-------	-99.57
	2015	821	4.90	+ 16.67	-93.88	0.12	-53.85	-99.80
	2016	991	7.40	+ 76.19	-90.75	0.31	+ 19.23	-99.48
	2017	1059	8.30	+ 97.62	-89.63	0.35	+ 34.62	-99.42
	2018	1141	5.10	+ 21.43	-93.63	0.10	-61.54	-99.83
	2019	1112	3.80	-9.52	-95.25	0.16	-38.46	-99.73
	2020	953	2.40	-42.86	-97.00	0.19	-26.92	-99.68

During 2016, the simulated 1-hour highest maximum ground level concentration of NO_2_ is 320.5 µg/m^3^ with insignificant increase by 6.833% as compared to the EEAA standards and 68.33% as compared to the base year. Also, it reached 82.9 µg/m^3^ for 24-hours, which represents an increasing by 55.24% than the base year, and still lower than EEAA standards by 44.73%. Moreover, the highest maximum ground level concentration of SO_2_ in 2016 for 1-hour and 24-hours exceeds the base year values and still lower than the EEAA standards. The concentrations of both NO_2_ and SO_2_ at 1-year during 2016 and 2017 are lower than EEAA standards but still greater than the base year. The NO_2_ and SO_2_ emissions at the main stack and their maximum concentration levels either at 24-hours or 1-year are often negatively correlated with TDF, RDF, and coal, while they often positively correlated with DSS, and diesel as shown in Table 9. Where, the NO_2_ has the highest positive correlation with diesel and DSS, while the highest positive correlation for SO_2_ is detected with DSS and natural gas. As well as, the NO_2_ has the highest negative correlation with TDF and natural gas, while the highest negative correlation for SO_2_ is detected with RDF and TDF. Increasing the percentage of diesel as a fuel lead to an increase in the emission of NO_2_ significantly at 1-hour with a correlation of 0.67, medium at 24-hours with a correlation of 0.35, and weakly during 1-year with a correlation of 0.19.

**Table 9:** Correlation between the fuel and both NO_2_ and SO_2_ emissions and maximum concentration levels throughout the period 2014–2020

Gas	Time-average	Natural gas	Diesel	Coal	RDF	DSS	TDF
NO_2_	1-hour	-0.13*	+ 0.67*	-0.14*	+ 0.03*	+ 0.42*	-0.81*
	24-hour	-0.08*	+ 0.35*	-0.01	-0.11*	+ 0.45*	-0.88*
	1-year	-0.20	+ 0.19	+ 0.19*	-0.10*	+ 0.39*	-0.71*
	Emission	-0.27*	+ 0.57*	-0.01*	+ 0.32*	+ 0.13*	-0.82*
SO_2_	1-hour	+ 0.39	+ 0.13	-0.32*	-0.70	+ 0.53*	-0.38*
	24-hour	+ 0.53	-0.09	-0.32*	-0.89*	+ 0.56*	-0.32
	1-year	+ 0.22	-0.17	+ 0.00*	-0.64*	+ 0.45	-0.23
	Emission	+ 0.27*	+ 0.09*	-0.20	-0.48*	+ 0.32*	-0.47*

* = statistically significant (*p*-value < 0.05)

Furthermore, an increase in DSS leads to an almost similar increase in NO_2_ emissions at the three time-scales (1-hour, 24-hours, and 1-year). Contrary, an increase in the percentage of TDF leads to a considerable decrease in NO_2_ emissions at the three time-scales. On the other hand, an increase in the percentage of coal and RDF lead to a slight decrease in NO_2_ emissions. An increasing in the percentage of DSS leads to a considerable increase in SO_2_ emissions and concentrations and conversely with both RDF, TDF, and coal over the three time-scales. According to Abouzeed et al. [1] the use of coal instead of natural gas to produce 60 million tons/year of cement leads to an increase in emissions of NO_2_ by 22.899 tons, SO_2_ by 221.796 tons, PM10 by 41.491 tons, PM2.5 by 38,103 tons and carbon emissions by 9,323,321 tons. These excess emissions lead to an increase in the added societal cost to the national economy of about 2.8–3.9 billion dollars annually.

Thus, it is clear that the increase in the percentage of both diesel (70.99%) and DSS (0.56%) together during 2016 leads to a considerable increase in the emissions concentrations of both NO_2_ and SO_2_ over the three time-scales. Therefore, increasing the percentage of TDF and RDF with decreasing the percentage of DSS, diesel will decrease the concentration of both NO_2_ and SO_2_ and enhance the ambient air quality. This is matched with Hekal et al. [15] findings which indicated that the use of alternative fuels, such as RDF, in the cement production reduces the amount of NO_2_ and SO_2_ emissions and leads to a significant reduction in the cost of energy. Therefore, the use of alternative fuels has a positive impact on the environment because it helps to get rid of solid waste and reduce gas emissions.

**Fig. 9:**
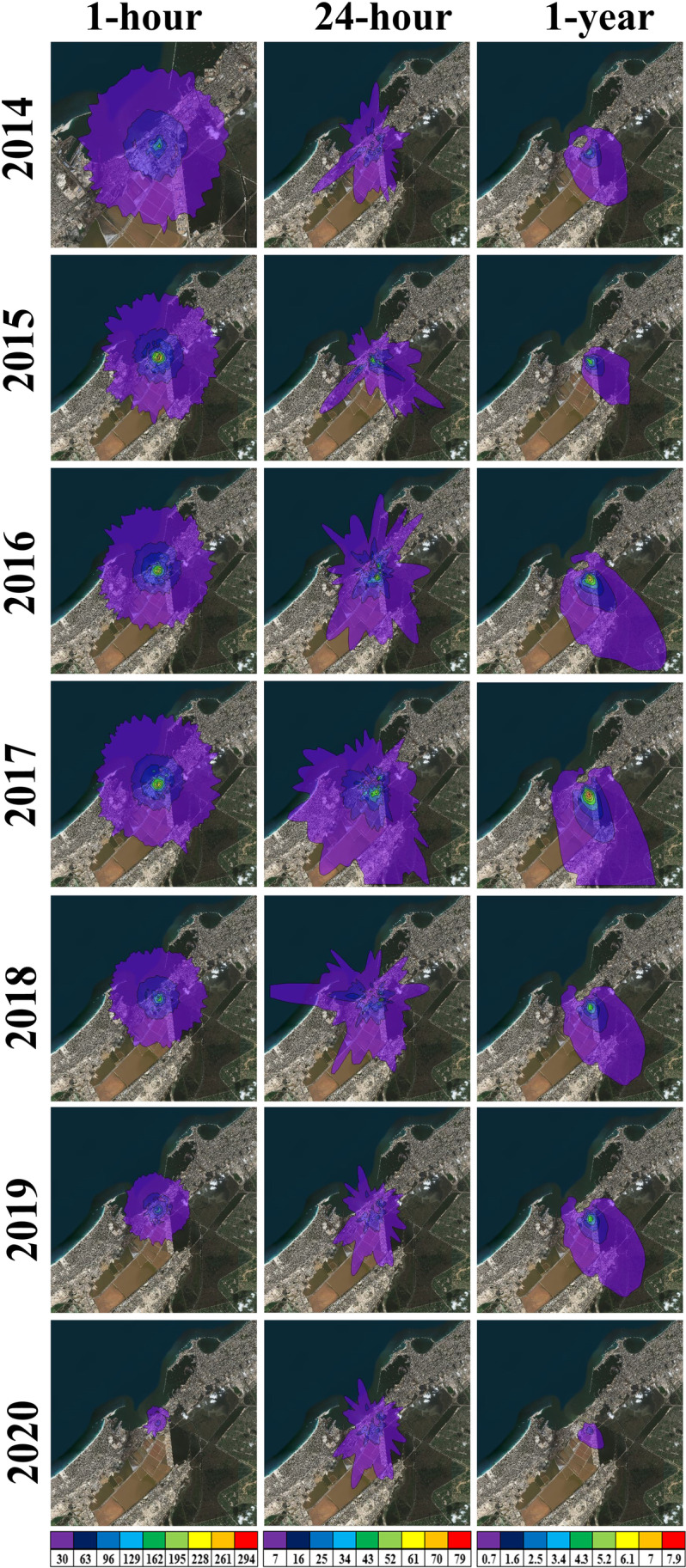
The predicted ground level concentrations (µg/m^3^) of NO_2_ at 1-hour (left panels), 24-hours (middle panels), and 1-year (right panels)

**Fig. 10:**
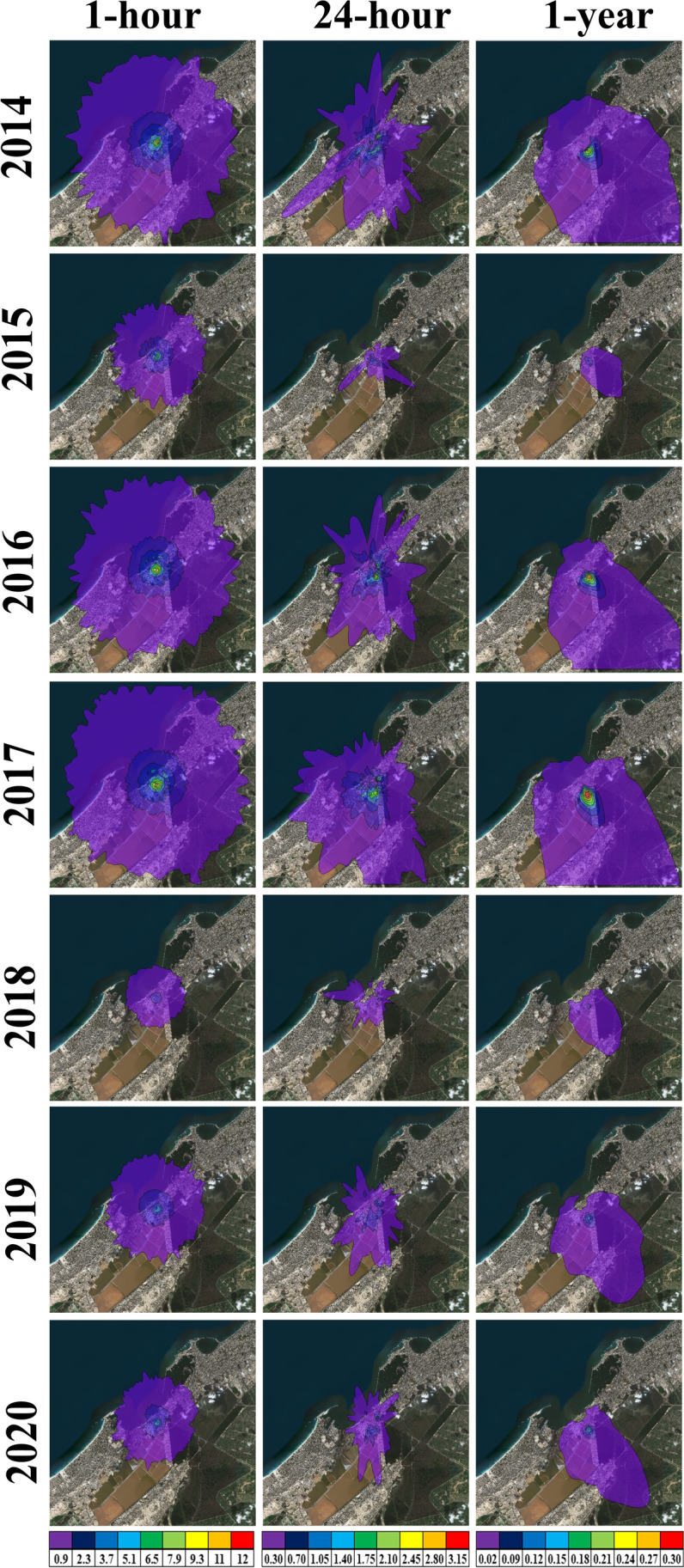
The predicted ground level concentrations (µg/m^3^) of SO_2_ at 1-hour (left panels), 24-hours (middle panels), and 1-year (right panels)

Figures 9 and 10 display the spatial distribution of the maximum concentrations of the dispersed NO_2_ and SO_2_ estimated from AERMOD at 1-hour, 24-hours, and 1-year during the period 2014–2020. The highest maximum concentrations of both NO_2_ and SO_2_ are mostly found southeast and south of the main stack, like TSP, and vary from year to another according to the intensity of wind speed. The closest distance between the APCC main stack and the receptor that has the maximum concentration of NO_2_ and SO_2_ throughout the study years occurred mostly in 2015 especially for 1-hour (236), 24-hours (390 m south), and 1-year (820 m south), due to the wind speed of 4 m/s is the mostly prevailing winds. Moreover, the farthest receptor with maximum NO_2_ and SO_2_ concentrations are found during the years from 2017 to 2019 for the three time-scales, where about 10% of the prevailing wind has speed of 10 m/s followed by 50% of 4 m/s wind speed. Generally, the simulated maximum concentrations of SO_2_ at the three time-scales are lower than the EEAA standard limits by at least 69.2%. Also, the NO_2_ has lower values as compared to EEAA standard limits except the greater value at 1-hour in 2016 with 20.50 µg/m^3^.

The lowest maximum concentration and dispersion of NO_2_ (Fig. 9) is detected during 2020, where it decreased as compared to the base year (2014) by -55.3%, -53.6%, and − 42.3% for 1-hour, 24-hours, and 1-year respectively, due to increasing the TDF percentage and decreasing the diesel percentage. While the NO_2_ has the highest maximum concentration and dispersion in 2016 and 2017 are due to presence of both diesel and DSS. For SO_2_, the preference year is 2018, as its percentage decreased to -67.6%, -56.4%, and − 61% for 1-hour, 24-hours, and 1-year respectively as compared to 2014 (Fig. 10), due to increasing the percentage of RDF and coal with absence of the DSS. While the SO_2_ has the highest maximum concentration and dispersion in 2016 and 2017 due to entrance of DSS as alternative fuel and decreasing the RDF percentage without TDF.

## Conclusion

In this study, the impact of using alternative fuels (Refuse Derived Fuels (RDF), Dried Sewage Sludge (DSS), and Tire-Derived Fuel (TDF)) as a mixture with coal in Titan Alexandria Portland Cement Company (APCC) for the cement industry on pollutant (dust/gas) emissions and ambient air quality was investigated and interpreted. The AERMOD dispersion model was used to simulate the concentration and dispersion of the Total Suspended Particles (TSP), nitrogen dioxide (NO_2_), and sulfur dioxide (SO_2_) at 1-hour, 24-hours, and 1-year averages. The simulations were performed on 4-domains multi-tier grids with 24,016 receptors over area of 20 km^2^ around the APCC main stack based on the observed emissions data from the APCC monitoring system during 2014–2020, meteorological data from the fifth-generation mesoscale model (MM5) and terrain elevation data from the USGS.

It is concluded that the change of fuel from natural gas during 2014 as a base year to coal mainly caused an increase in TSP dust, NO_2_, and SO_2_ emissions and concentrations. Also, the use of alternative fuels (TDF, DSS, and RDF) led to alternative variations in the emissions and concentrations of both dust and gases. The highest TSP concentration level was due to the increasing of coal percentage and presence of DSS with positive correlations, while its lowest level was due to the presence of the natural gas and increasing of diesel and TDF percentages with negative correlations. On the other hand, the simulated maximum concentrations of both NO_2_ and SO_2_ are below EEAA slandered limits except for NO_2_ in 2016 with a slight increase by 20.5 µg/m^3^ (6.833%). The lowest maximum concentration of NO_2_ was due to increasing the TDF percentage (negative correlation) and decreasing the diesel percentage (positive correlation), while its highest value was due to the presence of both diesel and DSS with positive correlations. Moreover, the SO_2_ concentration level increased due to the increasing of DSS percentage with positive correlation and decreased due to the increasing of RDF and TDF percentages with negative correlations. Consequently, increasing the percentage of TDF and RDF with decreasing the percentage of DSS, diesel, and coal will decrease the emissions and concentrations of TSP, NO_2_, and SO_2_ and enhance the ambient air quality. Then, the use of an alternative fuel by cement plants is environmentally and economically justified and will reduce the environmental pollution [7, 11], which is agreed with Chatziaras et al. [7] and Fadayini et al. [11]. Thus, it is recommended to increase the percentage of TDF and RDF and decrease the percentage of DSS, diesel, and coal in cement plants to maintain fossil fuel reserves, reduce air pollution levels, and improve ambient air quality providing substantial health benefits. The highest maximum concentrations of TSP, NO_2_, and SO_2_ were found southeast and south of the APCC and vary from year to another according to the intensity of wind speed. Where the nearest receptor to the main stack, which has the maximum concentrations of TSP, NO_2_, and SO_2_ is found in 2015 because the prevailing wind speed is mostly 4 m/s, while the farthest receptors for maximum concentrations are found in 2017, 2018, and 2019 because about 10% of the prevailing winds has a speed of 10 m/s followed by 50% of 4 m/s.

## Data Availability

The data supporting the findings of this article is included within the article.
